# Health Systems and Self-Reported Medication Adherence in Patients with Hypertension: A cross-sectional comparison of Jamaica and Colombia

**DOI:** 10.21203/rs.3.rs-7454001/v1

**Published:** 2025-09-05

**Authors:** Marshall K. Tulloch-Reid, Selena Lewis, Carene Lindsay, Siyi Geng, Paola Lanza, Jacqueline Duncan, Trevor Ferguson, Patricio Lopez-Jaramillo, Jose Lopez-Lopez, Farah Allouch, Lizheng Shi, Nadia Bennett, Gregorio Sanchez-Vallejo, Gustavo Aroca-Martinez, Jiang He

**Affiliations:** 1 The University of the West Indies, Mona, Jamaica; 2 Peter O’Donnell Jr. School of Public Health, UT Southwestern Medical Center, Dallas, Texas 75390, USA; 3 Masira Research Institute, Universidad de Santander (UDES), Bucaramanga, Colombia; 4 Facultad de Ciencias de la Salud Eugenio Espejo, Universidad UTE, Quito, Ecuador; 5 Department of Epidemiology, Tulane School of Public Health and Tropical Medicine, Tulane University, New Orleans, LA 70112, USA; 6 Fundación Cardiomet Cequin and Universidad del Quindio, Armenia, Colombia,; 7 Centro de Investigaciones en Ciencias de la Vida, Facultad de Ciencias de la Salud, Universidad Simón Bolívar and Departamento de Nefrología, Clínica de la Costa, Barranquilla, Colombia.

## Abstract

Robust health care systems can support medication adherence as a strategy to improve blood pressure control in Latin America and the Caribbean. The Caribbean and South American Team Based Strategies to Control Hypertension (CATCH) study, conducted in Jamaica and Colombia, allowed us to examine how differing health systems (Jamaica – manual, paper-based, government run vs Colombia - electronic systems utilizing government contracted providers) influenced self-reported antihypertensive medication adherence among hypertensive patients. A total of 576 hypertensive patients from 14 primary care clinics completed telephone interviews between August 2021 and February 2022 during the COVID-19 pandemic. Self-reported medication adherence was measured using the IMPACTS-MAS questionnaire and patients categorized as having high (6), medium (5–5.5) or low (<5) adherence based on score. Country was used as a proxy for health systems in multivariable logistic regression models with medium/low adherence as the primary outcome. Jamaican patients were more likely to report medium/low adherence (49.3% vs 11.8% p <0.001). In Colombia, younger (< 60 years) and never married patients reported more medium/low adherence. Jamaican patients experienced longer wait times for services but were more likely to discuss medication changes with the pharmacist while Colombian patients had more discussions with their doctor. Jamaicans had a higher odds of medium/low adherence compared to Colombians (OR: 6.78 (95% CI: 3.91, 11.75) after adjusting for sociodemographic factors and health care experiences. Further exploration of health system issues that may explain these differences can inform strategies to improve medication adherence in the region.

## Introduction

Hypertension is a major risk factor for cardiovascular disease, chronic kidney disease, and dementia (1, 2). It is estimated that hypertension is responsible for 8.5 million deaths globally, 88% of which take place in low and middle-income countries (LMICs) (2).

The 2016–17 National Jamaica Health and Lifestyle Survey reported a hypertension prevalence of 34% (blood pressure (BP) ≥ 140/90 mmHg and/or taking medications for hypertension), with only 59% of people with hypertension being aware of their diagnosis (3). Of those with diagnosed hypertension, 70% were on treatment but only 31% of those on treatment had a BP of ≤ 140/90 mmHg (3). In Colombia, the May Measurement Month Campaign conducted in 2019 reported a prevalence of hypertension of 28%, with approximately two-thirds of participants aware of their condition and 60% receiving antihypertensive treatment (4). Similar to Jamaica, 38% of those on treatment were controlled (4).

LMICs generally have poor BP control rates, despite the availability of effective medications for treating hypertension (5). Poor medication adherence, which includes failure to initiate treatment, take medications as prescribed, and maintain long-term therapy contribute to these low levels of BP control (6–8).

Several sociodemographic factors have been associated with poor medication adherence. These include male sex, younger age (under 60 years old), being single or unemployed, smoking, multi-morbidity, poor hypertension knowledge and depression (8–14). In countries such as United States, Canada, and the United Kingdom, medication adherence is lower in Black and Hispanic and other minority populations (14–16). While these factors can influence medication adherence, the health systems (all the organizations, institutions and resources that are responsible for managing health) also play a critical role in this outcome (14, 17). Inadequate access to healthcare, medication availability and accessibility, long waiting times and poor patient-provider communication have been associated with reduced medication adherence (17), However, use of fixed dose combination therapies, full prescription coverage and reduced copayments for healthcare services are associated with improved adherence (18, 19).

The aim of the Caribbean and South American Team Based Strategies to Control Hypertension (CATCH) study is to evaluate the implementation of a team-based strategy to improve hypertension management in Jamaica and Colombia. Both countries, located in the Latin America and Caribbean Region, with predominantly Hispanic / Black populations, have a high burden of hypertension with different systems for healthcare management (3, 20). In Jamaica 50% of the population accesses healthcare through government clinics and health facilities using paper-based health records and appointment systems. Care is free of charge at point of service. However, healthcare in Colombia is delivered through practitioners contracted by private institutions (or state-run institutions) that are contracted by government through insurance companies to provide care to patients and use electronic medical records and appointment systems. All Colombian citizens are enrolled in a public health insurance plan that provides certain basic services and this can be supplemented with other plans.

In this paper, we present data from a quantitative survey conducted as part of the needs assessment for the implementation of the CATCH Study. We explored self-reported medication adherence in both countries and evaluated whether health care delivery systems explained any observed differences, after adjusting for important sociodemographic confounders.

## Materials and Methods

### Recruitment and Data collection

The overall design of the CATCH study needs assessment has been previously described (21). Patients with hypertension ≥ 18 years old attending primary care clinics (10 in Jamaica and 4 in Colombia for at least a year were recruited between August 2021 and February 2022, during the COVID-19 pandemic. Jamaican patients were recruited consecutively from the clinic attendees on appointment days on which chronic disease visits were scheduled while Colombian patients were selected from an electronic medical records system database.

Questionnaires were administered by telephone after obtaining verbal consent in the native language of the participant. The questionnaire captured data on sociodemographic factors (age, sex, marital status, education), the presence of co-morbidities (diabetes, hypercholesterolemia, stroke, kidney disease, depression, anxiety) and hypertension treatment experiences including wait time for services such as registration, to see the nurse, doctor and to obtain medication (less than 30 minutes, 30–60 minutes, > 60 minutes) and whether medication choices, changes or substitution were discussed with the physician or pharmacists.

### Hypertension knowledge

Hypertension knowledge was evaluated using an 18-item questionnaire that included 10 questions about risk factors for hypertension (role of diet, exercise, sleep, emotions, family history), 6 on hypertension related outcomes (cardiovascular diseases, kidney disease and dementia) and 2 on aspects of treatment knowledge (use of medications and need for long term therapy).

### Medication Adherence Measurement

Adherence to antihypertensive medication was measured using the IMPACT- Medication Adherence Scale (MAS). The IMPACT-MAS scale consists of two questions: “Over the last 7 days, on how many days did you not take any blood pressure pills?” (Maximum score of 4 if no medications are missed with lower scores as the number of days increases, 0 if none were taken for all 7 days) and “Over the last 7 days, on how many days did you cut back (or not take the full dose) of your blood pressure pills?” (Maximum score of 2 if medications were taken as prescribed and 0 if reduction took places for all 7 days) with a total score calculated based on the response. Patients were classified as having high (Score = 6), medium (5 or 5.5) and low adherence <5 (22).

Ethical Approval for this study was obtained from the University of West Indies, the Ministry of Health and Wellness and Southeast Regional Health Authority Ethics Committees in Jamaica, Tulane University Institutional Review Board, USA and the Institutional Bioethics Committee of Universidad de Santander, Colombia.

### Statistical analysis

Weighted prevalence estimates were calculated for each country. Differences in percentages between Jamaica and Colombia were assessed using chi-square tests or the Fisher’s exact test depending on sample size. Medication adherence was the primary outcome of interest with characteristics of persons with high medication adherence (outcome =0) compared to those with low and medium medication adherence (outcome =1) in the logistic models. Country was used as a proxy for healthcare systems. Multivariable logistic models were adjusted for patient sociodemographic factors and patient experiences at their last clinic visit to determine the independent effects of healthcare delivery systems on medication adherence category. Odds ratios with 95% confidence intervals are presented for these effects.

## Results

Of the 576 hypertensive patients who were interviewed, 12 patients with hypertension who were not treated with antihypertensive medications were excluded from this analysis leaving a sample of 281 Jamaicans and 283 Colombians.

Table 1 presents the sociodemographic and health characteristics of the survey participants by country and medication adherence category (high vs medium/low). Jamaican patients were more likely to report medium/low medication adherence than Colombian patients (49.3% v. 11.8%, p <0.001). In Colombia, patients under 60 years (37% v. 18%, p = 0.044), who reported never having been married (26% v. 4.6%, p < 0.001) were more likely to report medium/low medication adherence. However, in Jamaica, none of the sociodemographic characteristics), were associated with self-reported medication adherence. In both countries, hypertension knowledge was not associated with medication adherence. In Jamaica, people with a history of heart attack/heart disease were less likely to report medium/low medication adherence (0.4% v. 6.0%, p < 0.001). None of the other medical conditions examined showed significant associations with medication adherence in either country.

### Healthcare Experiences that could impact Adherence

Patient experiences at their most recent clinic visit in each country are presented in Table 2. While a significantly lower percentage of patients in Jamaica compared to those in Colombia reported their doctor discussing their BP treatment plan, (56.8% v. 73.5%, p = 0.001), they were more likely to report their pharmacist discussing medication substitution (41.3% v. 7.8%, p <0.001). Jamaican patients experienced longer wait times (≥ 30 minutes), for registration (78.5% v. 16.6%, p <0.001), to see a physician (87.2% v. 7.7%, p <0.001) and to get their medication (81.2% v. 16.6%, p <0.001).

### Factors associated with Medication Adherence

The final multivariable logistic regression models used to explore the effect of health systems on the odds of low medication adherence are presented in Table 3. Jamaican patients were 7 times more likely to report medium/low medication adherence compared to Colombian patients after adjusting for age, sex, urban-rural status, education, and hypertension knowledge (OR: 6.78 (95% CI: 3.91, 11.75)). The odds of reporting medium/low medication adherence was inversely associated with age in the multivariable models. Differences in medication adherence were not explained by any of the healthcare experiences that patients reported on their last visit to the clinic. Besides country, age was the only other significant independent variable from multivariable analysis and was inversely associated with medium/low medication adherence. Other factors, including sex, urban-rural status, education level, hypertension knowledge and healthcare experiences did not show significant associations with medication adherence.

## Discussion

In our final multivariable analysis, after adjusting for age, sex, urban-rural status, education, and hypertension knowledge, Jamaican patients were found to be seven times more likely to report medium/low medication adherence compared to Colombian patients. The effect was not attenuated by any of the commonly reported confounders examined. Age was the only factor besides country that was inversely associated with low/medium medication adherence in multivariable models with a lower odds of having low/medium medication adherence with advancing age.

Jamaican patients experienced longer wait times for all clinic services. Medication adherence was not associated with patient experiences with health systems, and this did not explain country differences. In models that included experiences patients reported at the last clinical encounter; the findings were essentially unchanged. None of the individual experiences were independent determinants of medication adherence or able to explain country differences in medication adherence that we demonstrated.

In bivariate analysis age and marital status were found to be associated with medication adherence in Colombia only, with younger persons (< 60 years) and those who have never been married and widowed having medium/low medication adherence. These findings are consistent with factors reported to be associated with better medication adherence, specifically among Hispanics (23–25). However, while other studies have found other factors to be associated with low medication adherence (namely, hypertension knowledge, education level, hypertension complications, female gender, and being unemployed), we did not demonstrate these associations (26–28).

Patients in Jamaica with heart disease reported higher medication adherence, no associations were seen with other chronic health conditions. It is possible that patients with heart disease in Jamaica may receive additional support that helps improve adherence or that this condition may affect their perception of risk and improve adherence. We did not see this association in Colombia. Multimorbidity, which often results in increased pill burden and as a result poor medication adherence, was also not associated with medication adherence in this study (29).

Currently in Jamaica care for chronic diseases in Government run clinics may be limited to specific days of the week. Most clinics operate during working hours (8am to 4pm) and while dates for appointment are given, there is no specific time blocks and patients are seen in the order of arrival. Several measures are being initiated that may improve the long waiting times and potential delays in care, including extending opening hours at larger clinics and electronic health records system (to reduce time spent waiting for records to be retrieved). Additionally, the development of a National Health insurance scheme, similar to what exists in Colombia, could allow patients to access care through approved privately run health facilities which provide a more controlled environment that facilitates patients scheduling visits at more convenient times and obtaining care from a more consistent team of practitioners.

Medication distribution in Colombia is coordinated by the health insurance companies, who directly contract with medication providers and pharmacies for distribution. Patients are assigned to a pharmacy depending on insurance agreements with a specific provider and proximity to that facility. In some instances patients can also elect to select another local pharmacy for obtaining their medications if there is an agreement in place with their insurance company. A permanent audit system from the government ensures that national insurers fulfill their commitment to providing access and availability of medicines. While Jamaicans can access drugs from any of the government-run pharmacies with no fee at point of service, availability of prescribed medications and long wait times is often a challenge. Patients also have the option of purchasing their medications through private pharmacies using a government issued card which provides a subsidy on medications for specific chronic illnesses issues including hypertension. The government of Jamaica has instituted the Quick Prescript App that allows patients to submit their prescription by phone to the clinic pharmacies to determine the availability of medications in advance and reduce pharmacy wait (30).

Many approaches to healthcare delivery in Colombia (including the use of electronic health records system, a national health insurance scheme with universal coverage for chronic illnesses such as hypertension, allowing patients to access to a mix of private and public institution providers and health facilities) may also help to improve medication adherence in Jamaican patients. In addition, improving healthcare provider - patient communication in both settings may also improve medication adherence and hypertension control. Some of these issues will be addressed in the CATCH study which aims at improving hypertension control in both settings.

### Strengths and Limitations

Limitations of the study include the use of self-report for evaluation of medication adherence. We also did not measure BP or extract data from the medical records of respondents to determine the BP control. We cannot be certain as to whether any differences in adherence are related to cultural beliefs in both countries. The restrictions on face-to- face data collection due to the COVID-19 pandemic resulting in data collection by telephone did not allow us to ask additional questions that explored other issues affecting adherence such as transportation barriers including cost, safety concerns (particularly in the clinics in some inner-city communities), family support, polypharmacy and medication availability or access to combination therapy.

We included a representative sample of patients obtaining care in each setting which provided us with data to conduct this cross-country comparative study of hypertension care in Latin America and the Caribbean. We were able to adjust for known confounders reported from previous studies such as such as age, sex, and health insurance access to explore the independent effect of the health system on the outcome of interest.

## Conclusions

Understanding the health system factors that influence medication adherence is critical to improving hypertension control. Future research should include qualitative exploration of these findings and the evaluation of system factors that may contribute to these differences between Colombia and Jamaica. The CATCH study has been designed to address some barriers to medication adherence including addressing beliefs about BP and the medications used for treatment, communication with healthcare providers, team-based approaches to care and improving access to a simplified BP treatment algorithm with combination therapies. Systems change with engagement of providers, patients and administrators can strengthen medication adherence in both settings. Lessons from this experience will also be helpful to other LMICs and High Income Countries such as the United States, when optimizing hypertension treatment and control.

## Supplementary Files

This is a list of supplementary files associated with this preprint. Click to download.

- Table1JournalofHumanHypertensionFinal.pdf
- Table2JournalofHumanHypertensionFinal.pdf

## Data Availability

The original contributions presented in the study are included in the article; further inquiries can be directed to the corresponding author.

## Summary

### What is known

- Hypertension control in Latin American and the Caribbean remains suboptimal
- Medication adherence is an important determinant of blood pressure control and can be affected by several factors including systems of care

### What this study adds

- We found that Jamaican patients with hypertension were 7 times more likely to report low or medium self-reported medication adherence than Colombian patients.
- Country differences in medication adherence were not explained by known sociodemographic factors or patient health care experiences
- Use of electronic health records, a national health insurance policy and public private partnerships such as contracting clinical services through physicians’ offices may be important health system differences that explain country differences in medication adherence
